# Diagnosing Polyomavirus Nephropathy Without a Biopsy: Validation of the Urinary Polyomavirus-Haufen Test in a Proof-of-Concept Study Including Uromodulin Knockout Mice

**DOI:** 10.1093/infdis/jiae107

**Published:** 2024-03-01

**Authors:** Volker Nickeleit, Dalton Butcher, Bawana D Thompson, Lauraine H Rivier, Harsharan K Singh

**Affiliations:** Department of Pathology and Laboratory Medicine, Division of Nephropathology, University of North Carolina School of Medicine at Chapel Hill, Chapel Hill, North Carolina, USA; Department of Pathology and Laboratory Medicine, Division of Nephropathology, University of North Carolina School of Medicine at Chapel Hill, Chapel Hill, North Carolina, USA; Department of Pathology and Laboratory Medicine, Division of Nephropathology, University of North Carolina School of Medicine at Chapel Hill, Chapel Hill, North Carolina, USA; Department of Pathology and Laboratory Medicine, Division of Nephropathology, University of North Carolina School of Medicine at Chapel Hill, Chapel Hill, North Carolina, USA; Department of Pathology and Laboratory Medicine, Division of Nephropathology, University of North Carolina School of Medicine at Chapel Hill, Chapel Hill, North Carolina, USA

**Keywords:** biomarker, BK polyomavirus, haufen-test, transplantation, urine

## Abstract

**Background:**

Polyomavirus (PyV) nephropathy (PyVN) leads to kidney transplant dysfunction and loss. Since a definitive diagnosis requires an invasive kidney biopsy, a timely diagnosis is often hampered. In this clinical dilemma the PyV haufen-test, centering around the detection of 3-dimensional PyV aggregates in the urine, might provide crucial diagnostic information.

**Methods:**

A multistep experimental design was used. The hypothesis was that PyV-haufen form within the kidneys under high concentrations of uromodulin, a kidney-specific protein and that PyV-haufen are, therefore, kidney-specific disease biomarkers.

**Results:**

The first investigative step showed colocalization of uromodulin with aggregated PyV (1) in 10 kidneys with PyVN by immunohistochemistry, (2) in urine samples containing PyV-haufen by electron microscopy/immunogold labeling (n = 3), and (3) in urine samples containing PyV-haufen by immunoprecipitation assays (n = 4). In the in vitro experiments of the next step, only high uromodulin concentrations (≥1.25 mg/mL) aggregated PyV, as is expected to occur within injured nephrons. In contrast, in voided urine samples (n = 59) uromodulin concentrations were below aggregation concentrations (1.2−19.6 µg/mL). In the third investigative step, none of 11 uromodulin^−/−^ knockout mice (0%) with histologic signs of PyVN showed urinary PyV-haufen shedding, compared with 10 of 14 uromodulin^+/+^ wild-type mice (71%).

**Conclusions:**

PyV-haufen form within kidneys under high uromodulin concentrations. Thus, PyV-haufen detected in the urine are specific biomarkers for intrarenal disease (ie, definitive PyVN).

## Teaser

Our multistep proof-of-concept study demonstrates that the urinary polyomavirus (PyV)-haufen test is specific for PyV-induced end-organ kidney injury with lytic viral replication (ie, BKPyV nephropathy). Thus, the biomarker can now be incorporated into management guidelines for kidney transplant recipients.

In humans, latent asymptomatic polyomavirus (PyV) infections with BKPyV and/or JCPyV strains are commonly found in the urorenal tract. Viral disease with hemorrhagic cystitis or PyV nephropathy (PyVN) are seen only under immunosuppressed conditions, such as posthematopoetic stem cell or kidney transplantation. So-called definitive PyVN with end-organ disease is characterized by biopsy-confirmed histologic evidence of lytic intrarenal viral replication in tubules, tubular injury, tubulointerstitial inflammation, fibrosis, nephron loss, and renal dysfunction [1–3]. It is mainly caused by the BK virus strain (BKPyV) in kidney transplants, with a prevalence of approximately 5% in western countries. Since specific antiviral treatment is lacking and therapy rests on the reduction of overall immunosuppression, definitive PyVN with end-organ injury is clinically feared, with a graft loss rate of 8%–30% [2, 3].

To guide patient management, clinical risk assessment strategies for PyVN have been developed for kidney transplant recipients. They are based on laboratory assays detecting signs of BKPyV activation/replication by polymerase chain reaction (PCR) in plasma and urine, that is, BKPyV-DNAemia/viremia and BKPyV-DNAuria/viruria. However, these screening tests have only limited predictive values for an accurate diagnosis of definitive intrarenal end-organ disease. Definitive PyVN can occur with low viremia levels, and, vice versa, high viremia does not necessarily indicate viral nephropathy, as seen after hematopoietic stem cell transplantation [4]. Thus, PCR assays are not “kidney disease specific” and cannot be reliably used to diagnose definitive PyVN [5–8].

Currently, a diagnosis of definitive PyVN can be rendered only by a kidney biopsy. However, invasive biopsy procedures have limitations. Problems using biopsies occur in patients with bleeding disorders and other contraindications preventing an invasive approach. Expensive biopsy workups can also be challenging for underprivileged patients facing socioeconomic problems. Furthermore, problems arise in pharmaceutical anti-PyV drug trials, since renal biopsies are commonly not part of the study designs, and patient cohorts may not be adequately stratified into those with versus those without definitive PyVN.

Thus, the inherent shortcomings of PCR-based laboratory assays and the clinical limitations governing the use of invasive renal biopsies often hamper an optimal approach to patient management. Who has definitive PyVN and who does not?

We previously proposed a different method for diagnosing definitive PyVN. The so-called urinary PyV-haufen test (*haufen* is German for “heap” or “stack”) is based not on signs of PyV activation/replication but rather on specific structural abnormalities, that is, the detection of characteristic dense, 3-dimensional PyV aggregates in voided urine samples. These PyV-haufen are reportedly highly predictive for definitive PyVN [9–11]. However, the validity of the PyV-haufen test has not been fully established, since it remains undetermined whether PyV-haufen are “kidney-specific” markers.

The aim of the current study is to further validate the PyV-haufen test. We do so in a multistep approach. We test the hypothesis whether uromodulin, a kidney-specific glycoprotein also known as Tamm-Horsfall protein, secreted in the loop of Henle, is a crucial component for the formation of PyV-haufen. Uromodulin as an essential requirement for PyV-haufen formation would link the pathogenesis and clinical significance of haufen to observations made with other renal casts, i.e. dense aggregates of cells or proteins. Once shed into the urine, such casts serve as important well-established diagnostic markers in patients with kidney diseases.

In our current study we could prove that PyV-haufen detected in voided urine samples, are “castlike” and, indeed, organ/kidney specific. They can serve as diagnostic biomarkers for intrarenal PyV-induced injury with lytic viral replication—that is, definitive PyVN.

## METHODS

The study was approved by the University of North Carolina (UNC) Office of Human Research Ethics/Institutional Review Board (IRB nos. 05–1906, 08–1519, and 20–0293) and the Institutional Animal Care and Use Committee (ID no. 19-295.0). All rodent work was conducted with adherence to the National Institutes of Health Guide for the Care and Use of Laboratory Animals. All research involving human subjects was conducted with adherence to the Declaration of Helsinki, and the clinical and research activities being reported are consistent with the principles of the declaration of Istanbul as outlined in the Declaration of Istanbul on Organ Trafficking and Transplant Tourism. Urine and renal biopsy samples from humans were collected following local standard-of-care guidelines.

### Study Materials

A histologic diagnosis of definitive PyVN was established (or excluded) at time of biopsy at UNC according to Banff recommendations [3]. In the current study, all cases included as definitive PyVN carried a diagnosis of PyVN in Banff disease class 2, pvl score 2 or 3 [3, 12]. Leftover formalin-fixed and paraffin-embedded tissue/biopsy material was used for additional studies as needed. In selected clinical cases, standard diagnostic electron microscopy (EM) analyses were conducted at the time of allograft biopsy, and archived electron micrographs were reanalyzed for current study purposes.

Voided urine samples were collected from 3 UNC kidney transplant patient cohorts at the time of diagnostic kidney biopsy and based on the histologic biopsy diagnoses (clinical biopsy indications/clinical assumptions before biopsy included: suspicion of rejection, acute kidney injury, drug toxicity, recurrent renal disease, PyVN, and general signs of acute graft failure): (1) patients with PyVN Banff disease class 2, pvl score 2 or 3, viremia and viruria; (2) patients with viruria/shedding of decoy cells, varying levels of viremia but no corresponding histologic/biopsy evidence of definitive PyVN (ie, so-called PyV activators); and (3) kidney transplant recipients without viruria/viremia and no histologic/biopsy evidence of definitive PyVN. In addition a few urine samples were collected from healthy nontransplant volunteers. The urine samples were either fresh frozen or fixed in 2% paraformaldehyde for storage at 4°C. The isolation of PyV/PyV-haufen followed established protocols, including sequential centrifugation steps for clarification and concentration followed by negative staining and EM analysis (see Supplementary Data) [4, 9, 13, 14].

Black Swiss mice free of infection (Charles River Laboratories) and uromodulin (Tamm-Horsfall) knockout (KO) mice, strain 129/sv (kind gift of Dr Satish Kumar, MD, and James M. Bates, University of Oklahoma Health Sciences Center, Oklahoma City) were housed with access to food and water ad libitum. They were bred (Black Swiss outbred and 129/sv inbred) following general guidelines. Homozygosity in KO mice was monitored by genotyping and the absence of uromodulin further confirmed by enzyme immunsorbent assay (ELISA) testing of urine samples. All mice were infected by intraperitoneal injection of 50 µL of murine PyV strain A2 (containing 1–2 × 10^8^ viral gene equivalents by PCR or 1 × 10^5^ plaque-forming units/50 µL; A2 strain kindly provided by M. Fluck, PhD, Michigan State University), following a previously reported study design and protocol [15]. Infected mice were euthanized after 3–4 weeks when histologic evidence of intrarenal lytic viral replication/PyVN had developed.

Urine from mice voiding >40 µL was collected on parafilm and fixed in 2% paraformaldehyde for storage at 4°C. Subsequently, for EM grid preparation, between 80 and 200µL fixed mouse urine samples were centrifuged at 1500 rpm for 5 minutes to clear debris. Next, 30 µL of fluid from the bottom of the Eppendorf tube was discarded while the remaining supernatant was used for further analysis. Negative staining EM preparation to evaluate for the presence of murine PyV/murine PyV haufen followed established protocols [4, 9, 14]. (For protocols on histology and immunohistochemistry, see the Supplementary Data)

### Urinary PyV-Haufen Definition

In urine samples (human and mice), haufen were defined by EM as discrete aggregates of a minimum of 6 PyVs with a typical surface structure forming unequivocal dense 3-dimensional clusters (as reported elsewhere) [9, 10].

### Urinary PyV-Haufen Intertwined With Uromodulin: Analysis by EM and Immunogold Labeling

Three known PyV-haufen positive fresh frozen human urine samples were processed for EM analysis and immunogold labeling with an antibody directed against uromodulin, using established protocols (see Supplementary Data).

### Urinary PyV-Haufen Intertwined With Uromodulin: Analysis by Immunoprecipitation and Western Blotting

PyV-haufen were isolated by centrifugation of 4 fresh frozen urine samples from 4 patients with previously established haufen shedding (patient cohort 1 [see above]). Centrifuged urine from patients without biopsy-proven PyVN and previously confirmed lack of haufen shedding served as PyV-haufen negative control urine samples (n = 4; patient cohorts 2, 3, and urine from normal volunteers [see above]). Dynabeads were coated with rabbit anti-SV40PyV-VP1 antibody at 37°C overnight. For negative-bead control purposes, Dynabeads were coated with rabbit immunoglobulin G or remained uncoated. Then 30 µL of urine concentrates was incubated with the coated beads, and the eluates containing target proteins were subsequently saved. The immunoprecipitated protein complexes were separated by polyacrylamide gel electrophoresis (PAGE) and transferred to Immobilon (Millipore) membranes. The membranes were immunostained with a mouse monoclonal anti-PyV-VP1 capsid antibody, followed by an antibody directed against uromodulin. Chemiluminescence signals were captured on autoradiography films. PyV-capsid protein rendered a band in the 40–45-kDa and uromodulin in the 100–110-kDa range (see Supplementary Data for further details).

### Effect of Uromodulin Concentrations on PyV-Haufen Formation: An In Vitro Study

PyV (ie, free virions) were collected from fresh-frozen urine samples from 12 patients with viruria (BKPyV-DNAuria by PCR; range, 6.3 × 10^4^–3.7 × 10^6^ gene equivalents/mL), previously EM-confirmed urinary shedding of free virions without PyV-haufen, and no evidence of PyVN by renal biopsy (patient cohort 2 [see above]). Isolated free virions were resuspended in uromodulin-spiked aqueous buffer solutions mimicking intrarenal urine conditions in acutely injured tubules (test setting 1: sodium, 60 mmol/L; potassium, 80 mmol/L; calcium, 3.5 mmol/L; magnesium, 5 mmol/L; chlorine, 131 mmol/L; sulfate, 8 mmol/L; phosphate 10mmol/L; urea, 47 mmol/L; glucose, 18 mmol/L; pH 6.0; as described elsewhere [16]). In parallel (test setting 2), virions were resuspended in uromodulin-spiked urine aliquots collected from nontransplant healthy volunteers (see above). The final uromodulin concentrations in both sample sets after spiking ranged from 0.31 to 5.0 mg/mL. Negative controls were run in parallel, with albumin replacing uromodulin at corresponding concentrations or with samples not spiked with protein. Postincubation PyV and PyV-haufen formation were evaluated based on EM analysis, as described elsewhere [10, 14]. In each sample, PyV-haufen were recorded as either present or absent (see Supplementary Data for further details).

### Uromodulin Concentrations in Voided Human Urine Samples

Uromodulin concentrations were studied in 59 fresh-frozen urine samples from patient cohorts 1–3 (see above) by means of a sandwich ELISA technique using a modified protocol (see Supplementary Data) [17].

## RESULTS

### Histology of PyVN: Intratubular PyV Aggregation and Uromodulin Colocalization

**Figure 1.**
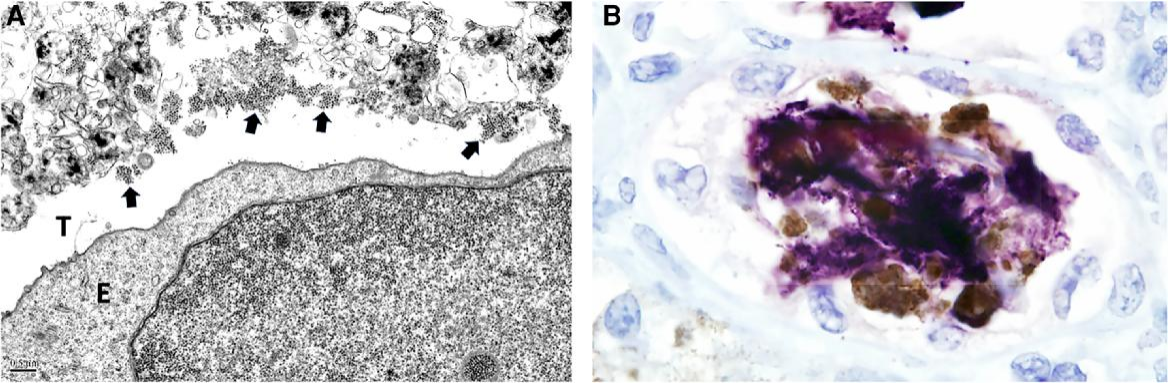
*A*, Electron microscopy (original magnification ×12 500). In polyomavirus nepthropathy (PyVN), lytic viral replication in tubular epithelial cells results in the release of viral progeny into tubular lumens (*T*), where they tend to aggregate and form small clusters (*arrows*); note injured tubular epithelial cell (*E*). *B*, Light microscopy (original magnification ×400). In PyVN, lytic viral replication results in the intratubular release of PyV progeny (*stained in brown*) that are seen in disintegrating epithelial cells, as well as in small extracellular clusters. Within tubules, virions are colocalized with uromodulin (stained in blue). Immunohistochemical double labeling was performed with antibodies directed against PyV-capsid protein (brown) and uromodulin (blue).

PyVs undergo lytic replication in renal tubular epithelial cells, leading to release of abundant virus progeny into injured tubular lumens, where they tend to aggregate (Figure 1*A*). Such dense clustering of PyV in acutely injured nephron segments is associated with uromodulin colocalization. This phenomenon was studied in 5 human and 5 Black Swiss mouse kidneys with PyVN. In all nephrons showing intratubular viral aggregates, uromodulin was also noted (Figure 1*B*).

### Urinary PyV-Haufen Intertwined With Uromodulin: Analysis by EM and Immunogold Labeling

PyV-haufen collected from voided urine samples showed aggregated virions intimately intertwined with uromodulin. This phenomenon was observed by EM and immunogold labeling of negatively stained urine samples (n = 3 patients; Supplementary Figure 1).

### Urinary PyV-Haufen Intertwined With Uromodulin: Analysis by Immunoprecipitation and Western Blotting

**Figure 2.**
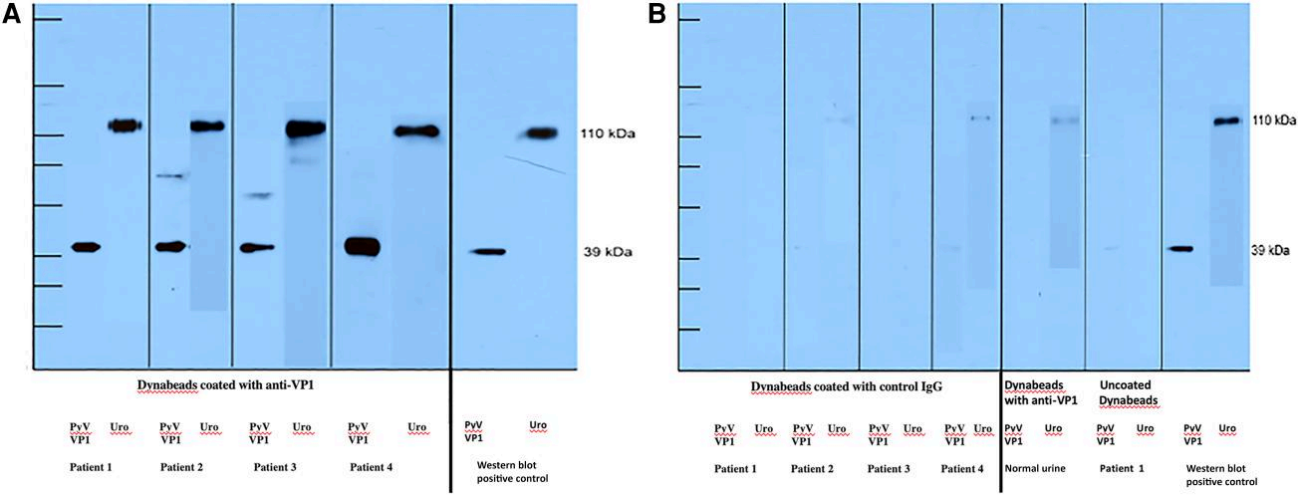
Immunoprecipitation and Western blots demonstrate tight colocalization of polyomavirus (PyV)­ capsid protein and uromodulin (Ur) in human urine. *A,* Test urine containing PyV-haufen (patients 1–4). PyV-capsid protein 1 (PyV-VP1) was isolated by immunoprecipitation. Dynabeads were coated with an antibody directed against PyV-VP1. Immunoprecipitated proteins (originating from the PyV-haufen) were subsequently separated by gel electrophoresis and visualized in Western blots showing strong bands in the expected 40–45-kDA range for PyV-VP1 and the 100–110-kDA range for the colocalized uromodulin. *B*, Control urine. In urine samples containing PyV-haufen (patients 1–4), immunoprecipitation with uncoated Dynabeads or Dynabeads coated with immunoglobulin G (IgG), followed by gel electrophoresis and Western blotting, did not reveal any specific signals for PyV-VP1 or uromodulin. In addition, a control normal urine sample did not show evidence of PyV-VP1 or uromodulin following immunoprecipitation with Dynabeads coated with anti–PyV-VP1 capsid antibodies.

PyV-haufen were isolated from urine samples of 4 patients with established PyV-haufen shedding by incubation with anti–PyV-VP1 antibody–coated magnetic Dynabeads. Samples from 4 patients without PyV-haufen shedding served as negative urine controls. Immunoprecipitated proteins were separated by gel electrophoresis, and VP-1 and uromodulin were visualized in Western blots. Incubation of corresponding urine samples with either immunoglobulin G–coated beads or uncoated Dynabeads served as negative-bead-controls. Urine collected from all patients with PyV-haufen shedding showed strong comigration of PyV-capsid protein 1 and uromodulin. No significant signals were seen in any of the controls (Figure 2).

### PyV Aggregation Promoted by High Uromodulin Concentrations: An In Vitro Study

In the urine uromodulin concentrations and polymerization vary depending on location (ie, bladder or intrarenal nephron segments) and tissue integrity (ie, normal or acutely injured tubules). The highest concentrations and most extensive polymerization are found in the kidney and in injured nephrons.

We studied the effect of various uromodulin concentrations on PyV aggregation/haufen-formation in 2 in vitro study settings (experimentally spiked samples): group 1, buffer conditions mimicking primary urine in injured nephron segments (acute tubular injury), and group 2, normal voided urine samples from healthy volunteers (n = 4 individuals, see above).

**Figure 3.**
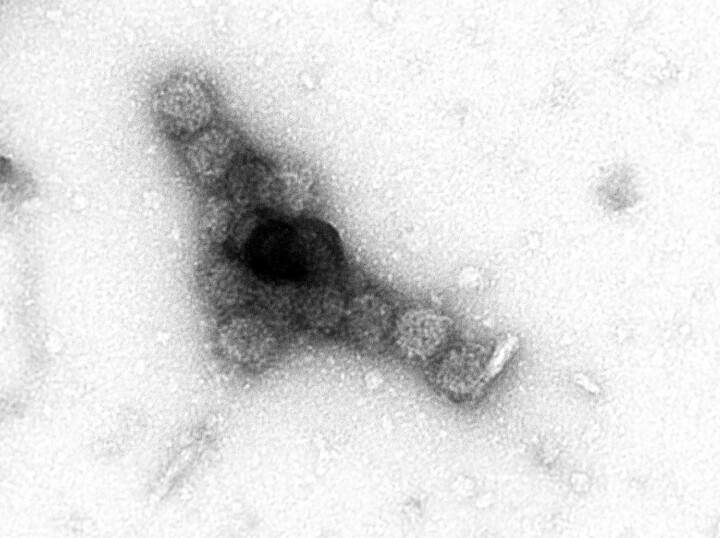
Negative staining electron microscopy (EM) in an in vitro experiment on polyomavirus (PyV) aggregation. A uromodulin concentration of 2.5 mg/mL promotes tight clustering of PyV in experimental buffer conditions mimicking, primary urine compositions in acutely injured nephron segments (transmission EM; original magnification ×100 000).

In both experimental conditions, external uromodulin spiking resulted in PyV aggregation/haufen formation. In the setting of acute tubular injury (group 1) PyV aggregation was detected at uromodulin concentrations as low as 1.25 mg/mL and higher (Figure 3). In comparison, in normal urine samples (group 2), only highest uromodulin concentrations resulted in PyV aggregation, best seen at 5.0 mg/mL. No aggregation was noted with low uromodulin concentrations or in all control samples in which uromodulin was replaced with albumin (Table 1). The background baseline virion density was comparable in all samples tested, based on the number of free virions detected by EM (median, 18–25 free virions per EM grid square; range, 10–30) indicating comparable test/PyV-aggregation conditions.

**Table 1.** Polyomavirus Aggregation Under Various Uromodulin Concentrations: An In Vitro Study^a^

Protein Added:	Protein Concentration, ng/mL	Test Condition of Urine	PyV Aggregation/Haufen Formation^b^	No. of Free Virions per Grid Square^c^
Uromodulin	5	ATI	Yes	21
		N	Yes	22
	2.5	ATI	Yes	22
		N	Very rare	20
	1.25	ATI	Yes	21
		N	No	18
	0.63	ATI	No	23
		N	No	22
	0.31	ATI	No	20
		N	No	23
Albumin	5	ATI	No	25
		N	No	23
	2.5	ATI	No	22
		N	No	20
	1.25	ATI	No	19
		N	No	22
	0.63	ATI	No	23
		N	No	24
	0.31	ATI	No	23
		N	No	22
None	NA	ATI	No	21
		N	No	23

Abbreviations: ATI, acute tubular injury like; N, normal voided urine; NA not applicable; PyV, polyomavirus.

^a^Uromodulin promotes PyV aggregation/haufen formation at high concentrations under ATI buffer conditions, as seen in injured nephrons; even higher uromodulin concentrations are required for aggregation in normal urine samples. These in vitro findings support an intrarenal origin of PyV-haufen formation with a predilection along tubules with active PyV replication and associated tissue injury (ATI).

Experiments were conducted in duplicate.

^b^Evaluated in 25 grid squares per electron microscopic grid per test sample.

^c^Free nonaggregated virions were counted in 25 grid squares per electron microscopic grid; listed are median numbers per grid square (range, 10–30 free virions per grid square). The number of free virions was comparable in each test sample and served as an overall control parameter for equal viral density in the entire cohort.

### Uromodulin Concentrations in Voided Urine Samples

Uromodulin concentrations were studied with ELISA in 59 voided urine samples from 42 patients: 3 patients (10 samples) with definitive biopsy-proven PyVN (patient cohort 1); 29 patients (29 samples) with PyV activation, ie, viruria/viremia, but no PyVN by biopsy (patient cohort 2); and 20 kidney transplant recipients (20 samples) with no viruria/viremia and no PyVN by biopsy (patient cohort 3). Median uromodulin concentrations in voided urine samples were as follows: definitive biopsy-proven PyVN, 4.5 mg/mL (range, 1.2–12.1 mg/mL); viremia/viruria but no PyVN, 4.6 mg/mL (0.5–17.1 mg/mL); no viremia/viruria and no PyVN, 7.3 mg/mL (3.5–19.6 mg/mL) (all differences not significant).

### Shedding of Urinary PyV-Haufen in Uromodulin KO Mice With PyVN

**Figure 4.**
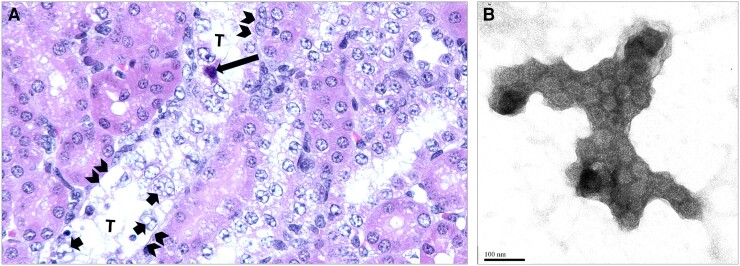
*A*, Light microscopy (hematoxylin-eosin stain; original magnification ×200). In a uromodulin^−/−^ knockout mouse with definitive polyomavirus nephropathy (PyVN), PyV replication in tubular cells has resulted in widespread injury, including nuclear enlargement and formation of type 4 (*short arrows*) and type 1 (*long arrow*) viral inclusions. Epithelial cell death and sloughing have resulted in segmental denudation of tubular basement membranes, that is, virally induced acute tubular injury (*arrowheads*); (*T* = tubular lumen). Note (not illustrated): identical histologic changes are seen in corresponding uromodulin^+/+^ wild-type (WT) mice with definitive PyVN. *B*, Negative staining electron microscopy (EM; original magnification ×100 000). In the urine from a uromodulin WT mouse with definitive PyVN, typical PyV-haufen with densely aggregated virions are found.

To investigate whether the formation and detection of PyV-haufen in the urine depend on the presence of intrarenal/intratubular uromodulin in vivo, we studied uromodulin^+/+^ wild-type (WT, n = 14) and uromodulin^−/−^ KO (n = 11) mice [18] with definitive PyVN (Figure 4*A*). In rodents, viral nephropathy with typical histologic signs of lytic replication can develop in 100% of animals after infection (personal observation; manuscript in preparation).

PyV-haufen were not noted in any urine samples of KO mice (0 of 11) by EM. In contrast, urinary PyV-haufen were detected in 71% of WT mice (10 of 14) (Figure 4*B* and Table 2). As expected in PyVN, all 25 mice (KO and WT) were shedding free polyoma virions in the urine as a positive “background control” for specimen adequacy [9, 14].

**Table 2.** Urinary Polyomavirus (PyV)–Haufen Shedding in Uromodulin^−/−^ Knockout and Uromodulin^+/+^ Wild-Type Mice With Histologic Evidence of PyV Nephropathy

Mice	Histologic Evidence of PyVN^a^	Urine Samples	
		PyV-Haufen^b^	Free Virions^c^
Uromodulin^−/−^ KO			
KO-1	Yes	Absent	Present
KO-2	Yes	Absent	Present
KO-3	Yes	Absent	Present
KO-4	Yes	Absent	Present
KO-5	Yes	Absent	Present
KO-6	Yes	Absent	Present
KO-7	Yes	Absent	Present
KO-8	Yes	Absent	Present
KO-9	Yes	Absent	Present
KO-10	Yes	Absent	Present
KO-11	Yes	Absent	Present
Uromodulin^+/+^ WT			
WT-1	Yes	Present	Present
WT-2	Yes	Present	Present
WT-3	Yes	Present	Present
WT-4	Yes	Present	Present
WT-5	Yes	Present	Present
WT-6	Yes	Present	Present
WT-7	Yes	Present	Present
WT-8	Yes	Present	Present
WT-9	Yes	Present	Present
WT-10	Yes	Present	Present
WT-11	Yes	Absent	Present
WT-12	Yes	Absent	Present
WT-13	Yes	Absent	Present
WT-14	Yes	Absent	Present

Abbreviations: KO, knockout mice; PyV, polyomavirus; PyVN, PyV nephropathy; WT, wild type.

^a^PyVN characterized by nuclear changes, lytic viral replication, acute tubular injury, and focal inflammation (see Figure 4*A*).

^b^Urinary PyV-haufen defined as ≥6 densely aggregated virions, according to previous reports [4, 10] (see Figure 4*B*).

^c^In PyVN, voided urine samples also show the shedding of free, nonaggregated virions; this feature serves as an internal nondiagnostic electron microscopic control for specimen adequacy, as previously reported [14].

## DISCUSSION

Reliable noninvasive diagnostic biomarkers are much-needed tools in the clinical repertoire, especially for diseases that commonly require invasive procedures for a definitive diagnosis. The diagnosis of definitive PyVN, as one example in this context, is conventionally based on histologic results from a kidney biopsy. PyV-haufen (ie, 3-dimensional tight aggregates of PyV found in voided urine samples) have previously been suggested as alternative noninvasive diagnostic biomarkers for definitive PyVN. However, further validation studies of the PyV-haufen test have never been conducted. Concern was raised regarding the organ specificity of the test. It was questioned whether haufen form within the kidneys or alternatively within the urinary bladder. Consequently, the clinical diagnostic use of the test was never generally endorsed.

In our current study, we aimed to prove the specificity of urinary PyV-haufen for intrarenal end-organ injury caused by lytic PyV replication in tubules. We focused on a kidney-specific protein—called *uromodulin* or *Tamm-Horsfall protein*—that is known to be an essential component of intrarenal cast formation. Is uromodulin also crucial for the genesis of PyV-haufen, and, consequently, can an intrarenal origin of urinary PyV-haufen be proved?

We showed in a first set of experiments that PyV-haufen were closely intertwined with uromodulin. In histologic sections of definitive PyVN, densely aggregated virions were found in close proximity with uromodulin in injured tubules. This association was also demonstrated by EM and immunogold labeling experiments on PyV-haufen extracted from the urine and by immunoprecipitation and Western blot studies: in haufen PyV and uromodulin were tightly colocalized. In a second in-vitro set of experiments, we demonstrated that PyV aggregated only in high uromodulin concentrations (≥1.25 mg/mL) and preferably in buffer conditions mimicking primary urine with high ionic strength, as found in injured tubules [16]. In comparison, in samples representing urine compositions in the urinary bladder, uromodulin concentrations were on average 100–1000 times lower than those required for PyV aggregation as established in the in vitro studies (with overall uromodulin concentrations detected in our voided urine samples similar to those in a previous report [19]).

These observations argued against the genesis of PyV-haufen in the urinary bladder. In the third in vivo set of experiments, the intrarenal origin of PyV-haufen was confirmed by using uromodulin^−/−^ KO mice with typical histologic features of a lytic intrarenal PyV infection/PyVN. Uromodulin^−/−^ KO mice with PyVN lacked urinary PyV-haufen shedding. In comparison, urinary haufen were found in 71% of uromodulin^+/+^ WT mice with PyVN. A discrepancy between previous studies on urinary PyV-haufen shedding (>90% in humans with PyVN [4, 9]) and current rodent data (71% in WT mice) is caused by technical challenges in collecting mouse urine.

Our experiments prove the intrarenal genesis of PyV-haufen. For viral aggregation within tubules with lytic replication, several promoting factors seem to act in concert: (1) high uromodulin concentrations in the thick ascending limb of Henle/distal nephron segments where uromodulin is secreted, (2) lytic PyV replication in distal nephron segments with high uromodulin concentrations, and (3) PyV-induced acute tubular injury and corresponding low intratubular flow of primary urine with high ionic strength [16, 20, 21]. We demonstrate that, indeed, the chain of events for PyV-haufen formation is similar to other forms of cast formation, such as myeloma casts or various cellular casts [22–25], that are all well-established biomarkers of intrarenal disease with uromodulin as a crucial building block. Consequently, castlike PyV-haufen found in voided urine samples serve as specific biomarkers for intrarenal disease—that is, definitive PyVN. PyV-haufen do not form in the urinary bladder.

Current recommendations for patients at risk of PyVN rest on the paradigm that individuals presenting with viral disease show laboratory signs of viral replication, such as viruria or viremia. However, vice versa, only a minority of patients with evidence of viral replication also present with end-organ-disease. Thus, diagnostic decision making is challenging since current clinical screening assays can identify patients at increased risk for end-organ disease, but they cannot unequivocally allow for a definitive diagnosis of PyVN. Consequently, additional studies, such as invasive kidney biopsies, are needed for diagnostic workup (reviewed in [26]). Because the urinary PyV-haufen test is based on the detection of specific structural changes (ie, the 3-dimensional aggregation of PyV), it differs from “replication-based” assays.

A qualitative PyV-haufen test in patient cohorts undergoing renal biopsy for various indications and clinical pre-biopsy assumptions, including rejection, drug toxicity, and suspicion of PyVN, and with varying levels of BKPyV-viremia, reportedly had >90% sensitivity, specificity, and positive and negative predictive values for a corresponding biopsy-proven diagnosis of definitive PyVN [9]. There was no concern about urinary PyV-haufen shedding in patients with clinically insignificant latent PyV infections. A quantitative PyV-haufen analysis showed tight correlations with the overall degree/severity of intrarenal lytic PyV replication (Spearman ρ = 0.85) and the Banff PyVN disease classes [4, 11]. (See Supplementary Data for more information on previously published clinical correlation studies and technical aspects of the PyV-haufen test.) Thus, taking the current “proof-of-concept” data into consideration, the urinary PyV-haufen test can now be regarded as a fully validated noninvasive biomarker for definitive PyVN.

**Figure 5.**
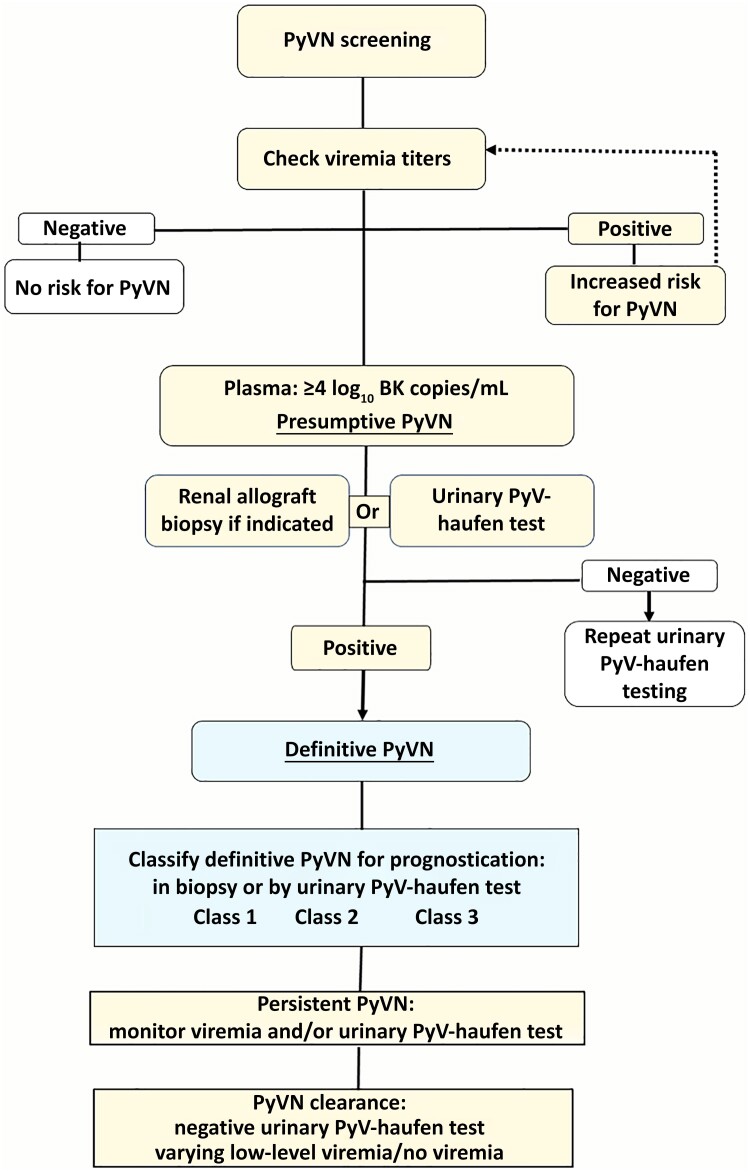
Polyomavirus nephropathy (PyVN) risk assessment and diagnosis in kidney transplant recipients. The flow chart illustrates recommendations for patient management, including the urinary PyV-haufen test as a now fully validated noninvasive diagnostic biomarker. BKPyV-DNAemia/viremia tested by polymerase chain reaction (PCR) assay; the differential diagnosis of a negative PCR test result includes false-negative PCR results due to mutant BKPyV strains, the presence of JCPyV or the presence of SV40PyV. Note: other renal diseases can concur with PyVN.

When should the urinary PyV-haufen test be used in patient management (Figure 5)? It is best suited as a targeted diagnostic tool in patients with signs of BKPyV replication based on currently recommended screening protocols—that is, in an established risk group of patients. In this patient cohort the urinary PyV-haufen test may replace a kidney biopsy for the diagnosis of definitive PyVN. This is of special importance if renal biopsy specimens cannot be easily collected, as in (1) patients with bleeding disorders, (2) some pediatric patients, (3) patients with socioeconomic challenges, (4) patients with a negative biopsy result but with concurrent and persistent signs of PyV replication/BKPyV-DNAemia requiring frequent repeat testing, or (5) patients with persistent PyVN, to monitor for signs of disease resolution [3, 8, 27, 28].

Furthermore, patients facing socioeconomic challenges and difficulties undergoing a costly biopsy procedure can especially benefit from a noninvasive urinary biomarker. In other instances, kidney biopsies are marginal and histologic results inconclusive. Here the PyV-haufen test can provide additional crucial diagnostic information. In addition, in pharmaceutical drug trials conducted to evaluate the efficacy of new antiviral agents, the test is of great diagnostic value, since biopsy procedures are not commonly part of the trial designs. Consequently, study groups are often not adequately stratified into patients with versus those without definitive PyVN, and data analysis of the trial results might be challenging [7].

In conclusion, we contend that PyV-haufen in voided urine samples are specific structural biomarkers for intrarenal lytic PyV replication—that is, definitive PyVN. These biomarkers can be used to diagnose PyVN noninvasively, to improve diagnostic accuracy, and to enhance a personalized approach to patient management. Based on the current proof-of-concept study, the urinary PyV-haufen test is now further validated and should be incorporated into recommendations and guidelines for the management of transplant recipients at risk of PyVN.

## Supplementary Data

Supplementary materials are available at *The Journal of Infectious Diseases* online (http://jid.oxfordjournals.org/). Supplementary materials consist of data provided by the author that are published to benefit the reader. The posted materials are not copyedited. The contents of all supplementary data are the sole responsibility of the authors. Questions or messages regarding errors should be addressed to the author.

## Supplementary Material

jiae107_Supplementary_Data

## Notes

***Author contributions***. All authors contributed to the study design, data interpretation, and preparation of the manuscript.

***Financial support*.** This work was supported by internal University of North Carolina sources only (including funding to pay the Open Access publication charges for this article).

## References

[1] Nickeleit V , Hirsch HH, Binet IF, et al Polyomavirus infection of renal allograft recipients: from latent infection to manifest disease. J Am Soc Nephrol 1999; 10:1080–9. 10232695 10.1681/ASN.V1051080

[2] Nickeleit V , Singh HK, Dadhania D, et al The 2018 Banff Working Group classification of definitive polyomavirus nephropathy: a multicenter validation study in the modern era. Am J Transplant 2021; 21:669–80. 32654412 10.1111/ajt.16189 PMC7891590

[3] Nickeleit V , Singh HK, Randhawa P, et al The Banff Working Group classification of definitive polyomavirus nephropathy: morphologic definitions and clinical correlations. J Am Soc Nephrol 2018; 29:680–93. 29279304 10.1681/ASN.2017050477 PMC5791071

[4] Nickeleit V , Davis VG, Thompson B, Singh HK. The urinary polyomavirus-haufen test: a highly predictive non-invasive biomarker to distinguish “presumptive” from “definitive” polyomavirus nephropathy: how to use it-when to use it-how does it compare to PCR based assays? Viruses 2021; 13:135. 33477927 10.3390/v13010135 PMC7833404

[5] Randhawa P , Kant J, Shapiro R, Tan H, Basu A, Luo C. Impact of genomic sequence variability on quantitative PCR assays for diagnosis of polyomavirus BK infection. J Clin Microbiol 2011; 49:4072–6. 21956980 10.1128/JCM.01230-11 PMC3232941

[6] Bateman AC , Greninger AL, Atienza EE, Limaye AP, Jerome KR, Cook L. Quantification of BK virus standards by quantitative real-time PCR and droplet digital PCR is confounded by multiple virus populations in the WHO BKV international standard. Clin Chem 2017; 63:761–9. 28100494 10.1373/clinchem.2016.265512

[7] Imlay H , Baum P, Brennan DC, et al Consensus definitions of BK polyomavirus nephropathy in renal transplant recipients for clinical trials. Clin Infect Dis 2022; 75:1210–6. 35100619 10.1093/cid/ciac071 PMC9525067

[8] Nickeleit V , Singh HK. Polyomaviruses and disease: is there more to know than viremia and viruria? Curr Opin Organ Transplant 2015; 20:348–58. 25933251 10.1097/MOT.0000000000000192 PMC4927320

[9] Singh HK , Andreoni KA, Madden V, et al Presence of urinary haufen accurately predicts polyomavirus nephropathy. J Am Soc Nephrol 2009; 20:416–27. 19158358 10.1681/ASN.2008010117 PMC2637054

[10] Singh HK , Donna Thompson B, Nickeleit V. Viral haufen are urinary biomarkers of polyomavirus nephropathy: new diagnostic strategies utilizing negative staining electron microscopy. Ultrastruct Pathol 2009; 33:222–35. 19895295 10.3109/01913120903241081

[11] Singh HK , Reisner H, Derebail VK, Kozlowski T, Nickeleit V. Polyomavirus nephropathy: quantitative urinary polyomavirus-haufen testing accurately predicts the degree of intrarenal viral disease. Transplantation 2015; 99:609–15. 25136849 10.1097/TP.0000000000000367 PMC4347732

[12] Loupy A , Haas M, Roufosse C, et al The Banff 2019 kidney meeting report (I): updates on and clarification of criteria for T cell- and antibody-mediated rejection. Am J Transplant 2020; 20:2318–31. 32463180 10.1111/ajt.15898 PMC7496245

[13] Biel SS , Nitsche A, Kurth A, Siegert W, Ozel M, Gelderblom HR. Detection of human polyomaviruses in urine from bone marrow transplant patients: comparison of electron microscopy with PCR. Clin Chem 2004; 50:306–12. 14684621 10.1373/clinchem.2003.024539

[14] Singh HK , Madden V, Shen YJ, Thompson D, Nickeleit V. Negative staining electron microscopy of urine for the detection of polyomavirus infections. Ultrastruct Pathol 2006; 30:329–38. 17090512 10.1080/01913120600932347

[15] Atencio IA , Villarreal LP. Polyomavirus replicates in differentiating but not in proliferating tubules of adult mouse polycystic kidneys. Virology 1994; 201:26–35. 8178487 10.1006/viro.1994.1262

[16] Wangsiripaisan A , Gengaro PE, Edelstein CL, Schrier RW. Role of polymeric Tamm-Horsfall protein in cast formation: oligosaccharide and tubular fluid ions. Kidney Int 2001; 59:932–40. 11231348 10.1046/j.1523-1755.2001.059003932.x

[17] Kobayashi K , Fukuoka S. Conditions for solubilization of Tamm-Horsfall protein/uromodulin in human urine and establishment of a sensitive and accurate enzyme-linked immunosorbent assay (ELISA) method. Arch Biochem Biophys 2001; 388:113–20. 11361126 10.1006/abbi.2000.2265

[18] Bates JM , Raffi HM, Prasadan K, et al Tamm-Horsfall protein knockout mice are more prone to urinary tract infection: rapid communication. Kidney Int 2004; 65:791–7. 14871399 10.1111/j.1523-1755.2004.00452.x

[19] Thornley C , Dawnay A, Cattell WR. Human Tamm-Horsfall glycoprotein: urinary and plasma levels in normal subjects and patients with renal disease determined by a fully validated radioimmunoassay. Clin Sci (Lond) 1985; 68:529–35. 3979015 10.1042/cs0680529

[20] Bachmann S , Metzger R, Bunnemann B. Tamm-Horsfall protein-mRNA synthesis is localized to the thick ascending limb of Henle's loop in rat kidney. Histochemistry 1990; 94:517–23. 2283315 10.1007/BF00272616

[21] Meehan SM , Kraus MD, Kadambi PV, Chang A. Nephron segment localization of polyoma virus large T antigen in renal allografts. Hum Pathol 2006; 37:1400–6. 16949647 10.1016/j.humpath.2006.06.016

[22] Ying WZ , Sanders PW. Mapping the binding domain of immunoglobulin light chains for Tamm-Horsfall protein. Am J Pathol 2001; 158:1859–66. 11337384 10.1016/S0002-9440(10)64142-9 PMC1891942

[23] Fairley JK , Owen JE, Birch DF. Protein composition of urinary casts from healthy subjects and patients with glomerulonephritis. Br Med J (Clin Res Ed) 1983; 287:1838–40. 10.1136/bmj.287.6408.1838 PMC1550059 6423037

[24] Yu CL , Tsai CY, Sun KH, et al Tamm-Horsfall glycoprotein (THG) is a binder for surface membrane proteins on blood cells and glomerular mesangial cells. Immunopharmacology 1997; 35:237–45. 9043937 10.1016/s0162-3109(96)00133-6

[25] Hoyer JR , Seiler MW. Pathophysiology of Tamm-Horsfall protein. Kidney Int 1979; 16:279–89. 393892 10.1038/ki.1979.130

[26] Kant S , Dasgupta A, Bagnasco S, Brennan DC. BK virus nephropathy in kidney transplantation: a state-of-the-art review. Viruses 2022; 14:1616. 35893681 10.3390/v14081616 PMC9330039

[27] Hirsch HH , Knowles W, Dickenmann M, et al Prospective study of polyomavirus type BK replication and nephropathy in renal-transplant recipients. N Engl J Med 2002; 347:488–96. 12181403 10.1056/NEJMoa020439

[28] Laskin BL , Singh HK, Beier UH, et al The noninvasive urinary polyomavirus haufen test predicts BK virus nephropathy in children after hematopoietic cell transplantation: a pilot study. Transplantation 2016; 100:e81–7. 26895217 10.1097/TP.0000000000001085 PMC4990513

