# Supplementary material for: Diagnosing Polyomavirus Nephropathy Without a Biopsy: Validation of the Urinary Polyomavirus-Haufen Test in a Proof-of-Concept Study Including Uromodulin Knockout Mice
Source: J Infect Dis. 2024 Mar 1;230(5):1120–9. doi: 10.1093/infdis/jiae107 (PMC11566231; doi:10.1093/infdis/jiae107)
Supplement: jiae107_Supplementary_Data [file jiae107_supplementary_data.docx]

**Supplementary Material**

Table of Contents ‘Supplementary Material’:

Pg 2: Supplementary Figure 1.

Pg 3-5: Additional Information “Methods”

Pg 6-8: Additional Information “Discussion”

Background Data: Comparative Studies of the PyV-haufen Assay in various Patient Populations, and Technical Considerations – previously published Reports briefly summarized.

Pg 8-9: References

**Supplementary Figure 1**


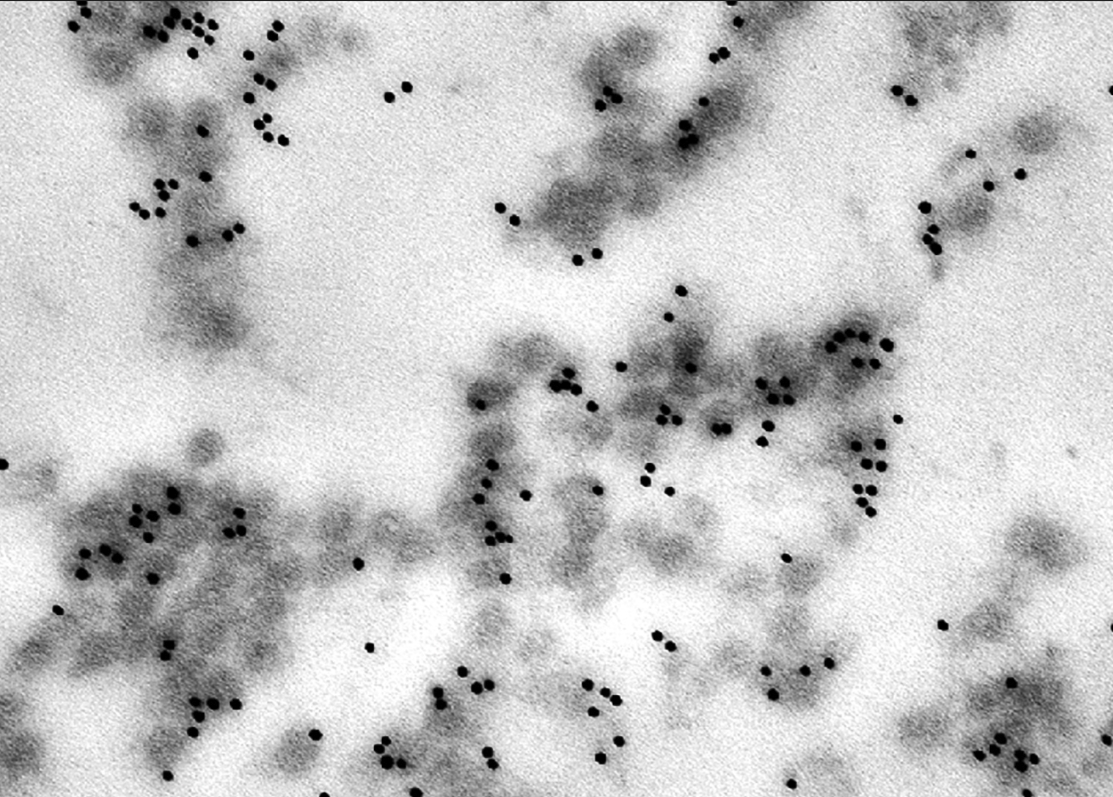


Human Urine: negative staining electron microscopy (EM) with immunogold labeling: A urinary PyV-haufen consisting of densely packed virions (gray dots) shows intertwined uromodulin (black beads). Transmission electron microscopy; immunogold labeling for uromodulin (black dots/beads with a diameter of 5 nm). Transmission EM, 100·000x original magnification.

**Additional Information “Methods”**

1. **Study Material**

Human urine samples were first cleared of large impurities such as cellular debris and membrane fragments by spinning the aliquots at low centrifugation (1500 rpm for 10 minutes) to pellet out the debris. The supernatant was subsequently filtered through a 5 µm filter to clear further debris and impurities. For isolation of *free non-aggregated virions*, cleared urine samples were subsequently ultra centrifuged at 36.000 rpm (100.000 g) for 60 minutes (used in the in-vitro experiments only). The *isolation of PyV-haufen* required ultra centrifugation at 12.500 rpm (20.000 g) for 35 minutes post clearing. Post ultra centrifugation virus containing pellets were collected. Free virions or PvV-haufen were analyzed by EM post negative staining using a previously described standard two-step methodology (adsorption followed by negative staining using 1% uranyl acetate) [1, 2]. PyV were identified by their characteristic ultrastructural size and morphology.

1. **Histology and Immunohistochemistry**

Formalin fixed and paraffin embedded tissue sections were used for histologic and immunohistochemical studies following standard protocols and antigen retrieval steps including microwaving in AR-10 buffer solutions for 4 minutes. In order to detect *PyV capsid protein-1 in human material* the mouse monoclonal Ab597 (cross reactive between BKPyV and JCPyV) was used at a dilution of 1:4000 (antibody kindly provided by Dr Jennifer Gordon, PhD, Lewis Katz School of Medicine, Temple University) followed by an anti-mouse envision plus ready-to-use detection system labeled polymer (Dako; cat#-K4001), DAB plus substrate chromogen (Dako; cat#-K3468) and chromogen/DAB enhancer (Dako; cat#-S1961). *In mouse tissue* *PyV capsid protein* was detected with a rabbit polyclonal anti SV40PyV- VP1 antibody (Abcam, cat# 53977) at a concentration of 1:2000 followed by incubation with an anti-rabbit envision plus ready-to-use detection system labeled polymer (Dako; cat#-K4003) and chromogen/DAB enhancer as listed above. *IHC for uromodulin (Tamm Horsfall protein) in human material* was conducted with a rabbit polyclonal antibody at a concentration of 1:1000 (Biomedical Technologies, cat# BT-590) followed by an anti-rabbit envision plus ready-to-use detection system (Dako; cat#-K4003) and chromogen/DAB enhancer as listed above. For double labeling experiments (anti uromodulin Ab followed by anti PyV-VP1 Ab) in humans, rabbit polyclonal anti uromodulin Ab incubation was followed by a biotinylated anti-rabbit IgG Ab (Vector; cat#-BA-100), ABC-alkaline phosphatase kit (Vector; cat#-AK-5000) and vector blue substrate as chromogen (Vector; cat#-SK5400). *IHC for uromodulin in mice* was conducted with a recombinant rabbit monoclonal anti-uromodulin antibody at a concentration of 1:2000 (Abcam, cat# EPR20071) followed by a biotinylated anti-rabbit IgG antibody (Vector Labs; cat#-BA-1000) and visualization with an ABC-alkaline phosphatase Kit (Vector Labs; Cat#-AK-5000) with vector red substrate (Vector Labs; SK-5000) as chromogen.

1. **Urinary PyV-haufen are intertwined with Uromodulin: Analysis by Electron Microscopy and Immunogold Labeling**

For immunogold EM labeling PyV-haufen collected from voided urine samples were stained with a monoclonal mouse anti-human uromodulin antibody, Cedarlane Laboratories cat# CL1032A and goat anti-mouse IgGFab’2, 5nm gold beads, British Biocell International/Ted Pella, Inc. cat# EMGFAF5) [3]. PyV/PyV-haufen were identified by their characteristic ultrastructural appearance and uromodulin by the detection of black dots/beads with a diameter of 5 nm.

1. **Urinary PyV-haufen are intertwined with Uromodulin: Analysis by Immunoprecipitation and Western Blotting**

Dynabeads (M280, Invitrogen cat# 142.03) were coated with rabbit anti-SV40PyV-VP1 antibody (Abcam cat# ab53977) or rabbit IgG (Santa Cruz cat# sc-2027; 1 mg of Dynabeads and 20 microgram antibody) at 37^0^ C overnight. Post centrifugation of previously classified urine samples from patient cohort#1 containing PyV-haufen, pellets were resuspended in 1mL Dulbecco’s PBS. As a first step, a 30 microliter aliquot was incubated with rabbit IgG coated Dynabeads at 37^0^ C for 60 minutes. Post this initial clearance and purification step, the collected eluates were subsequently re-incubated with rabbit anti SV40PyV-VP1 coated antibody beads. Controls: Post clearance eluates were (re)incubated with rabbit IgG coated beads or uncoated beads (instead of SV40PyV-VP1 antibody coating). In addition, eluates from previously classified urine samples not containing PyV-haufen (patient cohorts#2, #3, and normal, see above) were studied as negative urine controls. Following the second incubation, beads were washed in TBS/0.1% Triton buffer, placed into 2x Laemmli buffer (Thermo Fisher cat# J60015.AD), briefly boiled and centrifuged and the eluate containing precipitated target proteins from the Dynabeads saved. The immunoprecipitated complexes were separated by PAGE (9% acrylamide) and transferred to Immobilon membranes (Millipore cat# AIE-200). The membranes were immunostained with a mouse monoclonal anti PyV-VP1 capsid antibody Ab 597 (1:2000 in PBS-0.05% Tween 20; also see above) followed by a secondary goat anti mouse IgG (FAB)2-HRP antibody (Jackson ImmunoResearch Laboratories code# 115-036-003). Immunostaining was repeated with sheep anti-human uromodulin (1:6000, Sigma Aldrich, cat# AB733), followed by a rabbit anti-sheep IgG H&L (HRP) conjugate (Abcam cat# 6747). Chemiluminescence signals were captured on autoradiography films after development with SuperSignal West Pico PLUS Chemiluminescent Substrate (ThermoFisher cat# 34580). PyV-capsid protein rendered a band in the 40-45 kDa and uromodulin in the 100-119 kDa range. All experiments were performed in duplicate.

1. **Uromodulin Concentrations govern PyV-haufen formation: an in-vitro Study**

Ten mL urine per patient (n=12 patients; patient cohort#2 with viruria but lack of urinary PyV-haufen, see above) were ultra centrifuged at 100.000 g for 60 minutes and the pellets (approximately 300 microliter each) containing free, non-aggregated viruses used in the experiments. The viral pellets were batched (in order to generate a uniform stock solution) and 200 microliter aliquots containing free virions (confirmed by EM) subsequently resuspended in solutions spiked with various concentrations of uromodulin (0·31 – 5 mg/mL; incubation at 37^0^ Celsius for 60 minutes).

For current purposes PyV aggregation/haufen-formation was studied in two experimental settings: *group#1* buffer conditions mimicking primary urine in injured nephron segments (ATI); *group#2* normal (voided) urine from healthy volunteers (N, see above). In study group #2 four different urine samples were spiked and analyzed. For *negative control* purposes uromodulin was replaced with albumin or urine samples were not spiked. All individual experiments including resuspension and incubation steps were performed in duplicate.

Post incubation EM grids (one per test sample) were prepared with 30 microliter PyV containing test solutions. PyV and potential PyV-haufen formation were evaluated by negative EM staining as previously described.[4, 5] Per EM grid 25 squares were studied. PyV-haufen were recorded as either present or absent. In addition, free non-aggregated virions were counted per grid square and medians recorded. The number of free virions in the background served as overall control parameter for comparable viral density in the samples.

Uromodulin used for spiking experiments: lyophilized human glycoprotein, Cedar Lane/Fitzgerald, cat# 30R-AT051)

Albumin used for spiking experiments: human, fatty acid and immunoglobulin free, lyophilized albumin, Sigma-Aldrich cat# A3782

1. **Uromodulin Concentrations in voided human Urine Samples**

96-well plates were coated with sheep anti-human uromodulin (Sigma Aldrich, cat# AB733, 1:3000) followed by blocking with 1% BSA in PBS (both steps overnight at 4°C). Urine samples were diluted 1:200 and 1:400 in TEA buffer and tested in duplicate. The linear measurement range for the detection of uromodulin was 2-150 ng/mL. Standard curves (uromodulin, Fitzgerald cat#3OR-AT051) and negative buffer controls were included in each run. Uromodulin was detected with rabbit anti-human uromodulin (Biomedical Technologies cat# BT590, 1:10,000 in PBS/TWEEN) followed by goat anti-rabbit alkaline phosphatase conjugate (Sigma Aldrich cat# A3687, 1:20,000 in PBS/TWEEN). Signals were generated by pNPP- (para nitrophenil phosphate 1mg/mL in 1M diethanolamine buffer) and read at 405 nm in a Bio-Tek Analyzer.

**Additional Information “Discussion”**

**Background Data: Comparative Studies of the PyV-haufen Assay in various Patient Populations, and Technical Considerations – previously published reports briefly summarized.**

Previous reports together with data from the current proof-of-concept study underscore the diagnostic power of urinary haufen as proxy for PyVN, that is end organ kidney disease with lytic viral replication.

More than 1000 patients from various cohorts (kidney transplant recipients, non-transplant patients with native kidney diseases, patients post stem cell transplantation, few immunocompromised patients with definitive PyVN in native kidneys) were previously studied and data reported (retrospective analyses[6-8] and prospective analysis[9]). In these diverse patient populations, the previous studies focused on correlations of kidney biopsy findings with the presence of urinary PyV-haufen, BKPyV-DNAemia/viremia, and BKPyV-DNAuria or Decoy-Cell shedding/viruria (also see [10]). In addition, detailed protocols on sample collection, preparation, and test modalities were published[5, 9]. These studies showed:

- *Urinary PyV-haufen shedding was not tightly linked to viremia (kappa value 0.56) or viruria (kappa value 0.39).* Rather, PyV-haufen were only found in a subgroup of patients with viremia and viruria who also presented with corresponding biopsy confirmed definitive PyVN. Viremia in some patients with urinary PyV-haufen shedding and corresponding biopsy confirmed definitive PyVN was low (10E2 BKPyV-copies/mL plasma). In contrast, in some patients with high viremia (> 1x10E4 BKPyV copies/ml plasma) no urinary PyV-haufen and no biopsy evidence of definitive PyVN were found even after repeat testing.

Haufen shedding was not seen in the absence of viremia/viruria, and it was not found to be associated with common signs of kidney dysfunction including a diagnosis of ischemia induced acute tubular injury or kidney transplant rejection. There was no evidence that latent PyV infections lacking lytic cycles of viral replication were associated with any urinary PyV-haufen shedding.

- *Urinary PyV-haufen shedding showed a very strong correlation with histologic evidence of definitive PyVN*: qualitative urinary haufen test (absent/present) kappa value 0.98; quantitative urinary haufen test (number of haufen correlated with histologic degree of intra renal PyV replication) kappa value 0.86.
- *Urinary PyV-haufen shedding followed a dynamic pattern* during follow-up mirroring the disease course of definitive biopsy proven PyVN (from absent to positive to absent).
- Data from *quantitative urinary PyV-haufen tests correlated significantly with established Banff PyVN histologic disease* classes 1-3 (Banff 2019 update -Banff category 5[11]).
- The *sensitivity, specificity, negative and positive predictive values* for the qualitative urinary PyV-haufen test to detect definitive PyVN in a corresponding biopsy were: SE100, SP99, NPV100, PPV97 (corresponding values for plasma PCR test/viremia with cut-off 1x10E4 BKPyV copies/ml: SE72, SP88, NPV88, PPV74, respectively; urine PCR test/viruria with cut-off 1x10E7 BKPyV copies/ml: SE100, SP47, NPV100, PPV44, respectively).
- Urinary *PyV-haufen shedding* was noted in patients suffering from biopsy proven PyVN *post stem cell transplantation* (all with viremia and viruria) and few immunocompromised patients with *PyVN in native kidneys*. The overall test characteristics were very similar to those seen post kidney transplantation.
- The *interobserver reproducibility* rate of the urinary PyV-haufen analysis showed a kappa value of 0.90, concordance rate of 94.6%.
- *Shelf life*: Once fixed in 2% paraformaldehyde PyV,-haufen were stable for many months at 4^0^ Celsius. Fixed urine samples can be shipped at ambient temperature to reference laboratories.
- *Assay conditions*: Detailed protocols for sample collection, storage, preparation, testing, and analysis were published. The turn-around-time of the test was calculated to be around 3 hours.

Due to the fact that urinary PyV-haufen testing requires electron microscopy, test modalities are more complex compared to other (semi)automated techniques including PCR assays. Consequently, the urinary PyV-haufen test is not suited for mass screening. Rather, the haufen test should be used in a targeted manner in patients known to be at increased risk for PyVN and end-organ disease. Since PyV-haufen are very stable in urine fixed in 2% paraformaldehyde, samples can be stored and shipped to outside reference laboratories offering EM services as needed [6, 9]. Currently, efforts are underway to further develop technical assay conditions of the urinary PyV-haufen test with the goal to eliminate the need for EM analysis.

- *Costs*: A previous cost analysis listed net-expenses of approximately 300,-US$ that compared favorably with charges for PCR assays.
- *Diagnosing definitive PyVN by renal biopsy and the urinary PyV-haufen Test:* Small one-core kidney biopsies, such as those collected with an 18 gauge needle, can result in misdiagnoses of PyVN due to the focality of lesions. This problem is not unique for the diagnosis of definitive PyVN but also affects diagnoses of rejection etc.

The Banff working group on PyVN established recommendations for optimal/adequate biopsy samples to best diagnose PyVN histologically (optimal are 2 cores including medulla[11, 12]). In order to account for sampling issues, previous correlation studies comparing data from the urinary PyVN-haufen test with corresponding biopsy findings excluded small biopsy samples. Thus, the presence of urinary PyV-haufen can serve as proxy for definitive PyVN (further validated in the current study) and amend negative histologic PyVN diagnoses rendered in small/inadequate tissue samples.

As a general rule definitive PyVN is characterized by lytic intra renal viral replication with release of mature daughter virions into injured tubules and a microenvironment rich in uromodulin. This basic principle is not specific for PyVN in kidney transplants but also seen in native kidneys and PyVN observed in other species [13][14]. Since urine and urinary PyV-haufen are “secreted” end-products of the kidneys, they are excellent markers of intra renal injury.

In conclusion, previous reports in concert with the current proof-of-concept study underscore the diagnostic power, feasibility, and clinical usefulness of the urinary PyV-haufen test as a non-invasive specific biomarker for definitive PyVN. As with many tests, including currently used PCR assays for BKPyV, further standardization and validation studies are needed. Current efforts focus on offering the urinary PyV-haufen test in a kit format.

**References**

1. Hayat M, Miller S. Negative Staining. Vol. 1. New York: McGraw-Hill Publishing Company, **1990**.

2. Biel S, Gelderblom H. Electron microscopy of viruses. In: Cann A, ed. Virus cell culture - A practical approach. Oxford: Oxford University Press, **1999**:111-47.

3. Miller S, Howell D. Immunoelectron Microscopy. In: Howard G, Kaser M, eds. Making and Using Antibodies. Boca Raton, Florida: CRC Press; Taylor and Francis Group, **2007**:315-38.

4. Singh HK, Donna Thompson B, Nickeleit V. Viral Haufen are urinary biomarkers of polyomavirus nephropathy: New diagnostic strategies utilizing negative staining electron microscopy. Ultrastruct Pathol **2009**; 33:222-35.

5. Singh HK, Madden V, Shen YJ, Thompson D, Nickeleit V. Negative Staining Electron Microscopy of Urine for the Detection of Polyomavirus Infections. Ultrastruct Pathol **2006**; 30:329-38.

6. Singh HK, Andreoni KA, Madden V, et al. Presence of urinary Haufen accurately predicts polyomavirus nephropathy. J Am Soc Nephrol **2009**; 20:416-27.

7. Singh HK, Reisner H, Derebail VK, Kozlowski T, Nickeleit V. Polyomavirus nephropathy: quantitative urinary polyomavirus-Haufen testing accurately predicts the degree of intrarenal viral disease. Transplantation **2015**; 99:609-15.

8. Laskin BL, Singh HK, Beier UH, et al. The Noninvasive Urinary Polyomavirus Haufen Test Predicts BK Virus Nephropathy in Children After Hematopoietic Cell Transplantation: A Pilot Study. Transplantation **2016**; [100(10):e81-e87](https://journals.lww.com/transplantjournal/toc/2016/10000). doi:10.1097/TP.0000000000001085

9. Nickeleit V, Davis VG, Thompson B, Singh HK. The Urinary Polyomavirus-Haufen Test: A Highly Predictive Non-Invasive Biomarker to Distinguish "Presumptive" from "Definitive" Polyomavirus Nephropathy: How to Use It-When to Use It-How Does It Compare to PCR Based Assays? Viruses **2021**; 13:135.

10. Nickeleit V, Singh HK. Polyomaviruses and disease: is there more to know than viremia and viruria? Curr Opin Organ Transplant **2015**; 20:348-58.

11. Loupy A, Haas M, Roufosse C, et al. The Banff 2019 Kidney Meeting Report (I): Updates on and clarification of criteria for T cell- and antibody-mediated rejection. Am J Transplant **2020**; 20:2318-31.

12. Nickeleit V, Singh HK, Randhawa P, et al. The Banff Working Group Classification of Definitive Polyomavirus Nephropathy: Morphologic Definitions and Clinical Correlations. J Am Soc Nephrol **2018**; 29:680-93.

13. Sharma SG, Nickeleit V, Herlitz LC, et al. BK polyoma virus nephropathy in the native kidney. Nephrol Dial Transplant **2013**; 28:620-31. doi: 10.1093/ndt/gfs537.

14. Jennings SH, Wise AG, Nickeleit V, et al. Polyomavirus-associated nephritis in 2 horses. Vet Pathol **2013**; 50:769-74. doi: 10.1177/0300985813476063.
